# Community health workers as change agents in improving equity in birth outcomes in Detroit

**DOI:** 10.1371/journal.pone.0281450

**Published:** 2023-02-14

**Authors:** Jennifer K. Straughen, Jaye Clement, Lonni Schultz, Gwen Alexander, Yolanda Hill-Ashford, Kimberlydawn Wisdom

**Affiliations:** 1 Department of Public Health Sciences, Henry Ford Health System, Detroit, MI, United States of America; 2 Office of Community Health, Equity, and Wellness, Henry Ford Health System, Detroit, MI, United States of America; University of the Witwatersrand Faculty of Health Sciences / Pan African University, SOUTH AFRICA

## Abstract

We examined whether pairing pregnant women with community health workers improved pregnancy outcomes among 254 Black women with singleton pregnancies participating in the Women-Inspired Neighborhood (WIN) Network: Detroit using a case-control design. A subset (N = 63) of women were recontacted and asked about program satisfaction, opportunities, and health behaviors. Michigan Vital Statistics records were used to ascertain controls (N = 12,030) and pregnancy and infant health outcomes. Logistic and linear regression were used to examine the association between WIN Network participation and pregnancy and infant health outcomes. The WIN Network participants were less likely than controls to be admitted to the neonatal intensive care unit (odds ratio = 0.55, 95% CI 0.33–0.93) and had a longer gestational length (mean difference = 0.42, 95% CI 0.02–0.81). Community health workers also shaped participants’ view of opportunities to thrive. This study demonstrates that community health workers can improve pregnancy outcomes for Black women.

## Introduction

Racial disparities in pregnancy outcomes have persisted for decades, despite efforts to decrease these gaps. In the United States, Black women are almost twice as likely to have a low birth weight infant [1]. In addition, Black women have higher rates of preterm delivery (13.4%) when compared to their White counterparts (8.9%) [1]. These adverse pregnancy outcomes not only contribute to an increased risk of infant mortality [2], but a growing body of research suggests that infants born preterm or low birth weight have lifelong health consequences, including a higher risk of cardiovascular disease [3–5], diabetes [6], and other chronic diseases [7, 8]. As such, there is a critical need to develop and implement new strategies to improve pregnancy outcomes for minority women.

Historically, efforts to improve pregnancy outcomes have focused on increasing access to prenatal care and other services [9]. However, racial disparities in pregnancy outcomes are multifactorial, thus addressing gaps only in prenatal care discounts other contributors to racial disparities in pregnancy outcomes, such as stress, racism, social isolation, and poverty [10–12]. In Detroit and Michigan as a whole, these issues are particularly relevant as both have high rates of adverse pregnancy outcomes, especially among Black women [13]. For example, in Michigan, Black women have an infant mortality rate of 12.10/1000 live births whereas among White women it is 5.21/1000 live births [13]. According to the Michigan Department of Health and Human Services [14], infant mortality rates for Black women residing in Detroit are even higher (15/1000 live births in 2012–2014). Further, underserved women experience profound health consequences due to lack of steady employment, inadequate housing, insecure food sources, and other social factors that determine health, affecting their children as well [15–17].

In an effort to address these issues and the high infant mortality rates and disparities in pregnancy outcomes, four major, competing health systems in metropolitan Detroit committed their organizations to finding sustainable, collaborative solutions by forming the Detroit Regional Infant Mortality Reduction Task Force in 2008 (hereafter referred to as ‘the Task Force’). The Task Force is a public-private partnership including four competing health systems, three state and local health departments, two academic partners, and several non-government organizations. The Task Force established an effort called Sew Up the Safety Net for Women and Children, later rebranded as the Women-Inspired Neighborhood (WIN) Network: Detroit program to connect the right women to the right places at the right times to reduce racial disparities in infant mortality and the adverse pregnancy outcomes that contribute to the high infant mortality rates in Detroit. The WIN Network is an initiative designed to address social determinants of health to reduce infant mortality.

The WIN Network: Detroit uses an innovative and evidence-based approach [18–20] to initiate and sustain community engagement—creating greater opportunities for health education, goal-setting, and connection to safety net services. The goal is to empower women, promote the values of resilience and resourcefulness, and frame the program as a resource to support women in taking care of themselves and their families, all with the overarching program goal to reduce infant mortality and improve pregnancy outcomes. Additional objectives include training providers on healthcare equity and engaging non-pregnant women around pre- and interconception health, using a robust social marketing strategy. The key component to achieving these outcomes is the relationship-based model of incorporating community health workers (CHWs) in helping vulnerable pregnant women and women of reproductive age address both their medical and social needs. CHWs are frontline, entry-level workers who are uniquely qualified because of their life experience and skill-based training. In short, they provide personalized attention to each woman’s unique situation, history, and social needs and connect women to relevant resources that will help them have healthy pregnancies, healthy infants, and the hope of a more promising future for themselves and their families. In this analysis, we evaluate the effectiveness of the WIN Network CHWs at improving pregnancy outcomes and characterize WIN Network participants’ views of opportunities to thrive.

## Materials and methods

### Data collection

Pregnant women were eligible to participate in the WIN Network if they were between 18 and 39 years of age at the time of enrollment, self-identified as Black or African American, had at least one prior pregnancy, spoke English, and resided in Detroit. Women were excluded if they self-reported mental illness or substance abuse. The study was approved by the institutional review board (IRB) at Henry Ford Health (Detroit, MI) on May 23, 2012 under IRB #7252. All participants provided written informed consent. The WIN Network participants were recruited by CHWs at clinical and community sites, including obstetric appointments, Women, Infants, and Children (WIC) nutritional counseling (a federally funded program in the United States that provides nutrition counseling, nutritious food, breastfeeding support as well as referrals), pediatric appointments, and facilitated community events between 2012 and 2015. Participants received an intervention tailored to meet their individual needs, but included monthly or biweekly home visits by a CHW, referrals to local community resources, such as transportation, breastfeeding support, mental health services, insurance enrollment, family planning, housing, and food banks. Moreover, the CHWs provided mentoring, assistance, and support to promote a healthy lifestyle. Overall, 322 eligible pregnant women were enrolled in WIN Network (mean gestational age at enrollment = 24.2 weeks). For those women who had more than pregnancy while enrolled in the WIN Network, only information for their first pregnancy was included in this study.

The effectiveness of WIN Network at improving pregnancy outcomes was evaluated using a case-control design by comparing the birth outcomes of WIN Network participants (cases) to non-participants (i.e., the control population). Birth outcomes for WIN Network participants and controls were ascertained from linked birth-infant death vital statistics records collected by the State of Michigan. Of the 322 participants, 262 (81%) were linked to the state records using name, date of birth, and address. After linkage, an additional 8 women with a multiple pregnancy were excluded from this analysis. The final analytical sample included 254 women. The control population included Black women within the age range of the WIN Network participants who gave birth to a singleton infant during the same time period as the study participants (2012–2014). In addition, only women who resided in the same zip codes as the participants were eligible for inclusion. This resulted in a comparison population of 12,030 women. A subset of WIN Network participants were recontacted approximately 2 to 3 years after delivery of the index infant. A structured interview was used to collect data on maternal and child health behaviors as well as satisfaction with participating in the WIN Network. A total of 80 participants were reached. Seven declined to participate, 10 did not complete the interview, and 63 completed the follow-up interview.

### Statistical analysis

Data were analyzed using SAS version 9.4 (SAS Institute, Cary, NC). Sociodemographic and clinical characteristics of WIN Network participants and non-participants were compared using chi-square tests (for categorical variables). These variables included maternal age (18–20, 21–25, 26–30, and ≥ 31 years of age), education (less than high school, high school or general education equivalent), reported use of tobacco during pregnancy (yes/no), marital status (married, single, or widowed/divorced), and WIC use (yes/no). Maternal body mass index was calculated from the pre-pregnancy weight and height as recorded on the birth certificate. Body mass index was categorized as underweight (< 18.5 kg/m^2^), normal weight (18.5–24.9 kg/m^2^), overweight (25.0–29.9 kg/m^2^), and obese (≥ 30 kg/m^2^). Diabetes, gestational diabetes, chronic hypertension, gestational hypertension, and neonatal intensive care unit (NICU) admissions were classified as yes/no as reported in vital statistics records. Preterm birth was defined as delivery prior to 37 completed weeks of gestation and low birth weight included births that were less than 2500 grams. Infant death was defined as death occurring before one year of age. In addition, t-tests were used to evaluate birthweight and gestational age differences between WIN Network participants and controls when these variables were treated as continuous variables.

Odds ratios (OR) and 95% CIs for the association between WIN Network participation and categorical pregnancy and infant health outcomes were calculated using logistic regression. In these models, the predictors were WIN Network participation (yes vs no) and any potential confounding demographic or medical variables, while the dependent variables were low birth weight (yes vs no), preterm birth (yes vs no), NICU (yes vs no) and infant mortality (yes vs no). Those variables whose associations with WIN Network participation that had p-values <0.1 were considered as potential confounders and included in the adjustment models. Analysis of variance (ANOVA) models were used to assess the association between WIN Network participation and continuous outcomes of birth weight and gestational age. Similar to the logistic regression models, analysis of covariance (ANCOVA) models were also done to adjust for any potential confounders. The coefficients for these models would be the adjusted mean difference between the two groups. From these models, the mean difference or adjusted mean difference (either in birth weight [grams] or gestational age [weeks]) between the participant and the control groups were computed along with the corresponding 95% confidence intervals.

Although women who self-reported substance abuse or mental illness were not eligible to participate in WIN Network, we were unable to impose these restrictions on our control population as these data were not available. Therefore, as a sensitivity analysis, we excluded women in the control group who started prenatal care in the third trimester (N = 1107), had no prenatal care (N = 457), or were missing information about prenatal care or the month it started (N = 1296) as previous research suggests that women in these categories are more likely to have mental illness or suffer from substance abuse [21, 22]. In total, 9682 controls were included (80.5% of initial control population). Statistical significance for all analyses was assessed at the p ≤ 0.05 level.

## Results

The distributions of maternal sociodemographic and clinical characteristics by WIN Network participation are presented in Table 1. The WIN Network participants and controls were similar in terms of maternal age, education, and chronic disease prevalence. The WIN Network participants were more likely to report using WIC; 86.6% of WIN Network participants used WIC whereas 72.5% of the control population used WIC. When compared to the control population, a greater proportion of WIN Network participants (26% vs 21%) used tobacco, although the difference did not quite reach statistical significance (p = 0.055).

**Table 1:** Sociodemographic and clinical characteristics of WIN Network participants and controls.

Characteristic	WIN Network Participants (N = 254) N (%)	Controls (N = 12030) N (%)	p-value
Age (years)			
18–20	49 (19.3%)	2554 (21.2%)	0.713
21–25	99 (39.0%)	4642 (38.6%)	
26–30	63 (24.8%)	2670 (22.2%)	
31+	43 (16.9%)	2164 (18.0%)	
Education			
Less than high school	66 (26.0%)	2919 (24.3%)	0.493
High school or GED	107 (42.1%)	4824 (40.1%)	
Post-high school	78 (30.7%)	4108 (34.2%)	
Unknown	3 (1.2%)	179 (1.5%)	
Marital status			
Never married	231 (90.9%)	10376 (86.3%)	0.104
Married	21 (8.3%)	1477 (12.3%)	
Divorced/widowed	2 (0.8%)	164 (1.4%)	
Unknown	0 (0%)	13 (0.1%)	
Body mass index			
Underweight	5 (2.0%)	373 (3.1%)	0.278
Normal weight	91 (35.8%)	3647 (30.3%)	
Overweight	56 (22.1%)	2751 (22.9%)	
Obese	77 (30.3%)	3910 (32.5%)	
Unknown	25 (9.8%)	1349 (11.2%)	
WIC			
Yes	220 (86.6%)	8723 (72.5%)	<0.001
No	25 (19.8%)	2671 (22.2%)	
Unknown	9 (3.5%)	636 (5.3%)	
Tobacco use			
Yes	66 (26.0%)	2522 (21.0%)	0.055
No	187 (73.6%)	9430 (78.4%)	
Unknown	1 (0.4%)	78 (0.7%)	
Diabetes			
Yes	2 (0.8%)	101 (0.8%)	0.996
No	234 (92.1%)	11778 (97.9%)	
Unknown	18 (7.1%)	151 (1.3%)	
Gestational diabetes			
Yes	3 (1.2%)	356 (3.0%)	0.122
No	233 (91.7%)	11523 (95.8%)	
Unknown	18 (7.1%)	151 (1.3%)	
Chronic hypertension			
Yes	4 (1.6%)	270 (2.2%)	0.554
No	232 (91.3%)	11609 (96.5%)	
Unknown	18 (7.1%)	151 (1.3%)	
Gestational hypertension			
Yes	7 (2.8%)	325 (2.7%)	0.830
No	229 (90.2%)	11554 (96.0%)	
Unknown	18 (7.1%)	151 (1.3%)	

GED, General Education Development; SD, standard deviation; WIC, Supplemental Nutrition Program for Women, Infants, and Children; WIN, Women-Inspired Neighborhood

Table 2 presents the associations between WIN Network participation and infant health outcomes. The proportion of infants born low birth weight was lower among WIN Network participants (9.5% vs 11.6% in controls, p = 0.285), but the difference did not reach statistical significance. Birth weight was also evaluated as a continuous variable. Mean birth weight was quantitatively greater among WIN Network participants (3145.3 grams vs 3090.4 grams), but no statistically significant differences were detected (mean difference = 54.8, 95% CI -21.9–131.6, p = 0.161). The WIN Network participants had a lower rate of preterm births (13.4%) than controls (16.8%), but the difference did not reach statistical significance (OR = 0.77, 95% CI 0.53–1.10, p = 0.152). However, when gestational age was considered as a continuous variable, WIN Network participants had a significantly longer gestational length (mean difference = 0.42, 95% CI 0.02–0.81, p = 0.039). Infant mortality rates were similar between WIN Network participants (0.4%) and controls (1.5%). The WIN Network participants were about half as likely to have an infant admitted to the NICU (OR = 0.55, 95% CI 0.33–0.93, p = 0.025). After adjusting for the potential confounders of tobacco and WIC use, the difference between the two groups remained significant for NICU (OR = 0.54, 95% CI 0.32–0.94, p = 0.028) but not for gestational age as a continuous variable (adjusted mean difference = 0.31, 95% CI -0.08–0.70, p = 0.130).

**Table 2:** Association between WIN Network participation and infant health outcomes.

Infant Health Outcomes	WIN Network Participants (N = 254)	Controls (N = 12,030)	OR (95% CI) p-value	OR^1^ (95% CI) p-value	OR^2^ (95% CI) p-value
Low birth weight					
Yes	24 (9.5%)	1398 (11.6%)	0.79 (0.52,1.21) 0.285	0.79 (0.52, 1.21) 0.279	0.84 (0.54, 1.30) 0.430
No	230 (90.6%)	10629 (88.4%)			
Unknown	0 (0%)	3 (0%)			
Preterm birth					
Yes	34 (13.4%)	2017 (16.8%)	0.77 (0.53, 1.10) 0.152	0.77 (0.53, 1.10) 0.152	0.81 (0.56, 1.17) 0.265
No	220 (86.6%)	10001 (83.1%)			
Unknown	0 (0%)	12 (0.1%)			
NICU					
Yes	15 (5.9%)	1212 (10.1%)	0.55 (0.33, 0.93) 0.025	0.55 (0.33, 0.94) 0.028	0.54 (0.32, 0.94) 0.028
No	239 (94.1%)	10633 (88.4%)			
Unknown	0 (0%)	185 (1.5%)			
Infant mortality					
Yes	1 (0.4%)	175 (1.5%)	0.27 (0.04, 1.92) 0.189	0.28 (0.04, 1.99) 0.202	0.34 (0.05, 2.43) 0.280
No	253 (99.6%)	11855 (98.6%)			
	Mean ± SD	Mean ± SD	Mean Difference (95% CI) p-value	Mean Difference^3^ (95% CI) p-value	Mean Difference^4^ (95% CI) p-value
Birth weight (grams)	3145.3 ± 560.9	3090.4 ± 618.7	54.8 (-21.9, 131.6) 0.161	57.9 (-18.1, 95.1) 0.135	50.2 (-26.8, 127.2) 0.201
Gestational age (weeks)	38.7 ± 2.8	38.3 ± 3.2	0.42 (0.02, 0.81) 0.039	0.39 (-0.002, 0.78) 0.050	0.31 (-0.08, 0.70) 0.130

OR, odds ratio; SD, standard deviation; WIN, Women-Inspired Neighborhood; NICU, Neonatal intensive care unit; OR^1^, odds ratio adjusted for tobacco use, OR^2^ Odds ratio adjusted for tobacco use and WIC use. Mean difference^3^, mean difference adjusted for tobacco use, Mean difference^4^, mean difference adjusted for tobacco use and WIC use.

In order to determine whether gestational age at enrollment in the WIN Network was an important predictor of infant health outcomes, we conducted additional analyses among the participants only. Specifically, we stratified the participants by trimester of enrollment in the WIN Network and examined the proportion of participants with each health outcome within each strata (Table 3). The proportion of low birth weight infants was similar across trimester of enrollment; however, the proportion of preterm births and the proportion of NICU admissions increased as gestational age at enrollment increased. Thus, participants who enrolled in the WIN Network in the third trimester had higher rates of preterm birth (16.7%) than their counterparts who enrolled in the first (9.7%) or second trimesters (11.4%). Similarly, NICU admissions were lowest for participants who enrolled in the first (3.2%) or second (4.8%) trimesters as compared to those who enrolled in the third trimester (8.3%). However, none of these differences were statistically significant. Infant mortality was not included in this sub-analysis because only one participant was affected.

**Table 3:** Pregnancy outcomes by trimester of enrollment in WIN Network.

Pregnancy Outcome	Trimester of Enrollment			p-value
	First (N = 31) N (%)	Second (N = 105) N (%)	Third (N = 108) N (%)	
Low birth weight				
Yes	3 (9.7%)	10 (9.5%)	10 (9.3%)	0.996
No	28 (90.3%)	95 (90.5%)	98 (90.7%)	
Preterm birth				
Yes	3 (9.7%)	12 (11.4%)	18 (16.7%)	0.427
No	28 (90.3%)	93 (88.6%)	90 (83.3%)	
Neonatal intensive care unit				
Yes	1 (3.2%)	5 (4.8%)	9 (8.3%)	0.426
No	30 (96.8%)	100 (95.2%)	99 (91.7%)	
Birth weight (grams), mean ± SD	3081.1 ± 657.9	3144.9 ± 602.2	3170.3 ± 501.2	0.740
Gestational age (weeks), mean ± SD	38.5 ± 3.7	38.6 ± 2.9	38.7 ± 2.3	0.954

SD, standard deviation; WIN, Women-Inspired Neighborhood

In sensitivity analyses excluding women in the control group who had late or no prenatal care or were missing information about prenatal care, we found that the magnitude and direction of the observed effects were generally similar to what was observed when all controls were included, but somewhat attenuated (S1 Table). Notably, there remained a statistically significant decrease in NICU admissions in WIN Network participants as compared to controls (OR = 0.57; 95% CI 0.33–0.96). In addition, when compared to controls, WIN Network participants remained significantly more likely to use WIC (86.6% vs 75.8%, p < 0.001).

**Fig 1:**
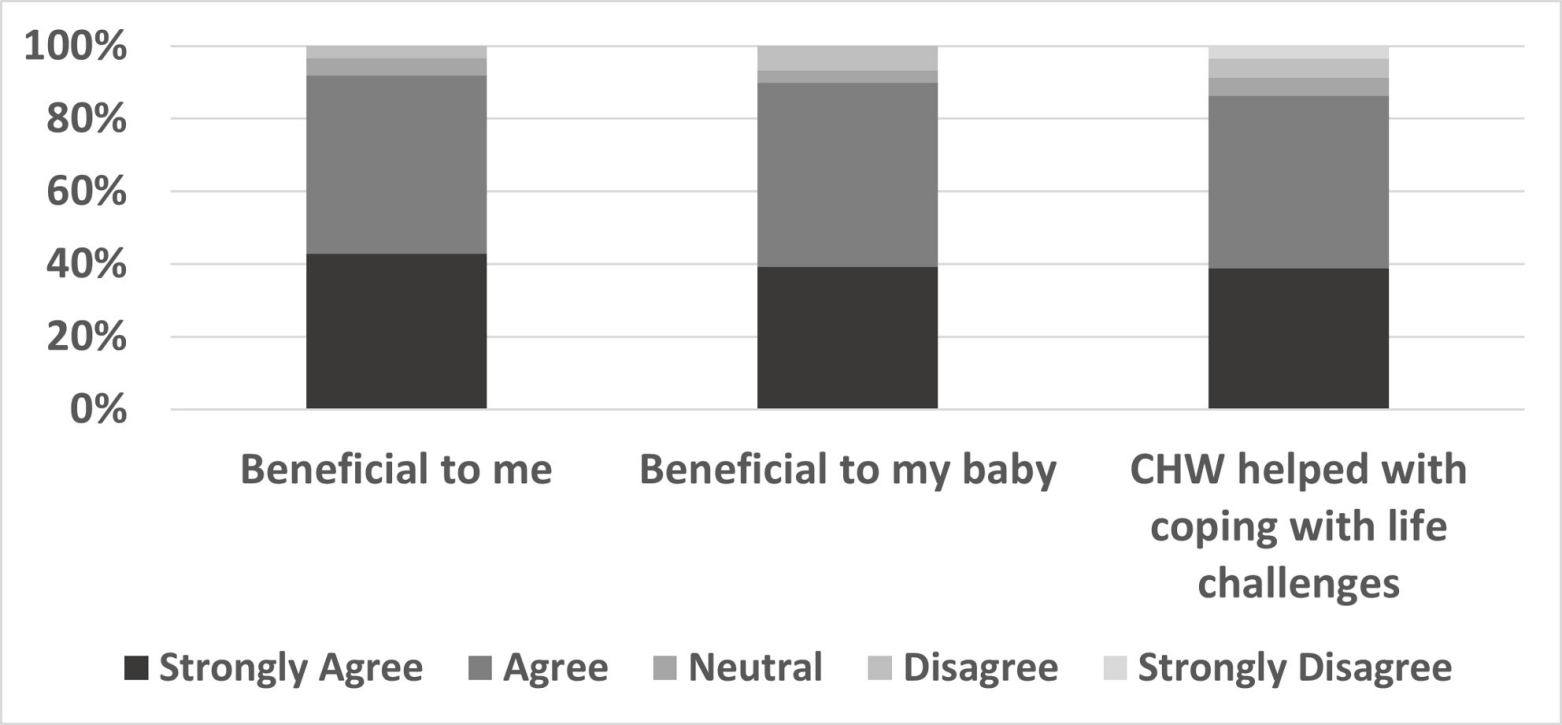
Summary of WIN Network participants’ views of participating in WIN Network and how their community health worker (CHW) helped 2–3 years after participating.

**Fig 2:**
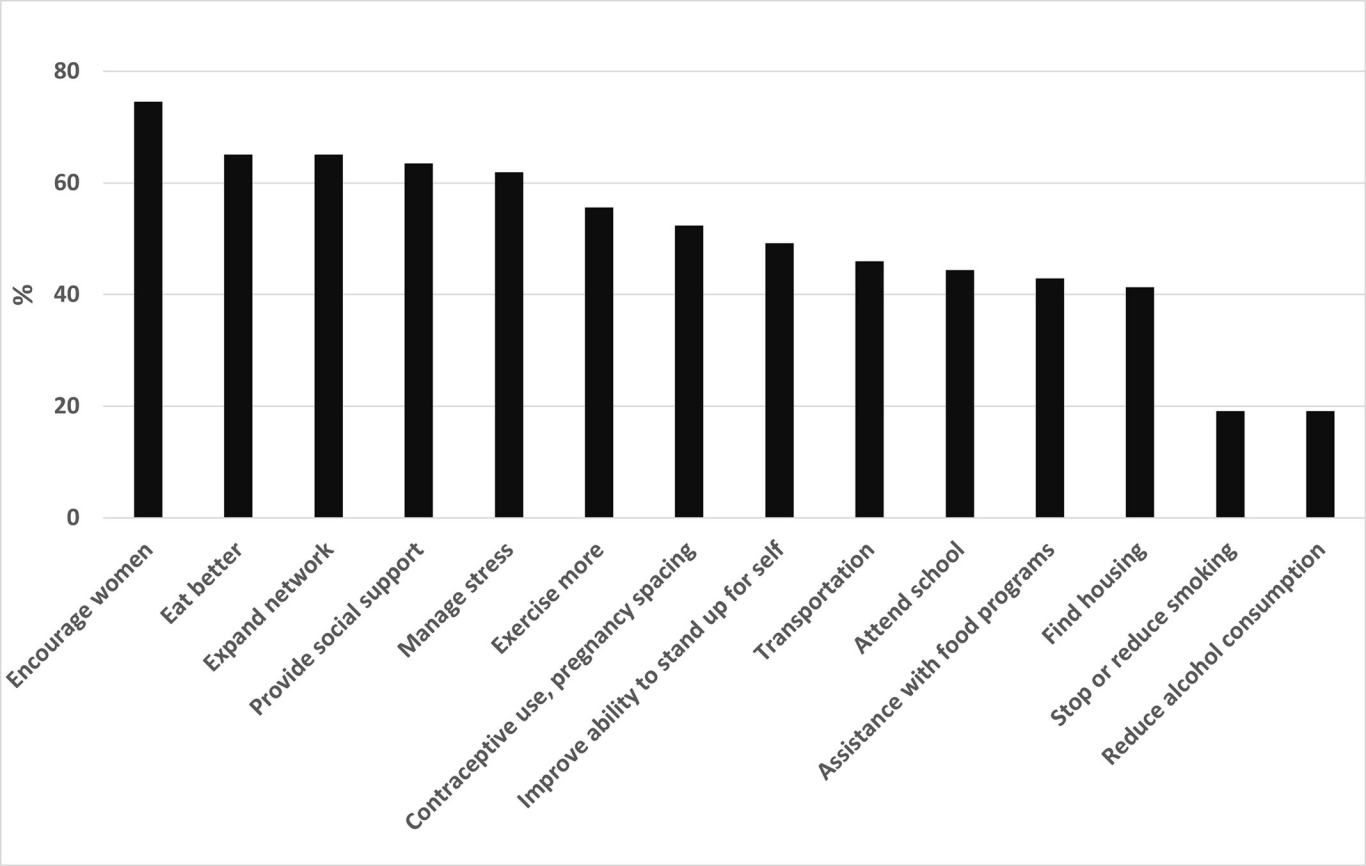
Summary of WIN Network participants’ views of how the program helped them with opportunities to thrive 2–3 years after participating.

Figs 1 and 2 summarize results of the WIN Network participant follow-up questionnaire. The majority of the women who completed the questionnaire agreed that WIN Network was beneficial to themselves (92.1%) and their infant (87.3%). Also, 80.9% of WIN Network participants reported that CHWs helped them feel more capable of coping with life challenges. The majority of women also reported that participation in WIN Network positively affected their lives by helping them attend school (44.4%), get a job (49.2%), and find housing (41.3%) and transportation (46.0%).

WIN Network also promoted healthy behaviors for the women (e.g., decreased alcohol use, decreased or stopped smoking, and increased exercise). In terms of psychosocial health, participants felt that WIN Network helped provide social support (63.5%), assisted with managing stress (61.9%), and helped improve their ability to stand up for themselves (49.2%). Participants also reported that WIN Network helped them with contraceptive use and pregnancy spacing (52.4%).

## Discussion

In this analysis of birth outcomes for urban area African American women, we found that participation in the WIN Network was associated with a significantly decreased admissions to the NICU and potentially longer gestational length, although this association became non-significant in adjusted models. In addition, WIN Network participants reported that participation benefited health behaviors and psychosocial health. These findings underscore the benefits of using CHWs to positively influence social determinants of health and improve pregnancy outcomes for Black women.

Our findings are in broad agreement with other studies that suggest that CHWs are influential in improving pregnancy outcomes among women at high risk of adverse pregnancy outcomes [18, 23, 24]. However, these studies used different strategies for developing a comparison group and incorporated CHWs into their interventions in slightly different ways. Similar to this study, Sabo et al. also found that integration of community health workers into ongoing programs (Health Start Programme) improved pregnancy outcomes as ascertained from vital statistics records; however, that study did not focus specifically on Black women [24]. Although WIN Network was not specifically developed to decrease NICU admissions, it is an important finding not only because NICU admissions are associated with significantly greater health care costs [25, 26], but also because previous studies have found that parents of infants admitted to the NICU have a higher prevalence of postpartum depression or anxiety and depression [27, 28]. These findings were corroborated by Masten et al. in a study that used community health workers to provide enhanced care during the pre- and postnatal periods [29]. Similar to our study, they also reported a decreased risk of NICU admissions as well as lower preterm birth rates among participants [29]. Together, these studies support the use of community health workers in prenatal care to potentially improve pregnancy outcomes for minority and high risk women.

Although the reason for the higher rate of WIC use among WIN Network participants as compared to controls cannot be substantiated, CHWs were trained to connect women to this type of resource, thus it is plausible that increased use is due to WIN Network participation. Our follow-up survey supports this idea as it indicated that CHWs were successful at connecting participants to food programs; 43% of respondents indicated that the WIN Network helped them with food programs. Nonetheless, we cannot eliminate the possibility that participants had a higher baseline need than controls and were therefore more likely to qualify for WIC.

Women could enroll in the WIN Network at any gestational age, thus some participants may not have been enrolled in the WIN Network for a sufficient length of time or they may not have enrolled early enough in their pregnancy to confer benefit.

As a sensitivity analysis we examined the infant health outcomes of WIN Network participants by trimester of enrollment. The findings suggest that increased duration of enrollment is associated with improved infant health outcomes, including fewer NICU admissions and fewer preterm births. The small sample size precluded formal evaluation of statistical significance, thus additional studies are needed to better characterize the effect of duration of enrollment on maternal and infant health outcomes. It is also important to note that we did not have complete data on the number of contacts each participant had with the CHW. The program is designed to meet the individual needs of the women, thus we would expect that the number of referrals and contacts with CHWs would vary greatly among participants. We are currently collecting the necessary data to evaluate the number of contacts and referrals needed in relation to the level of need as part of another study that is integrating CHWs into prenatal care.

Despite the suggestive findings of this analysis, several limitations merit mention. First, given the sample size of the WIN Network participants we may have been underpowered to detect statistically significant effects for preterm birth and low birth weight, despite lower prevalence of these outcomes among WIN Network participants. With the current ratio of WIN Network participants to controls and the observed control rates for the outcomes, odds ratios of 0.64 or lower (or 1.55 or higher) could have been detected with 80% power, assuming two-sided testing and alpha of 0.05. As mentioned above, we also could not estimate the dose response of the intervention because of incomplete data collection on the number of CHW home visits or events attended by participants. Additional, larger studies will be needed to further evaluate the effectiveness of CHWs in decreasing the rates of these adverse pregnancy outcomes. In addition, working with a low-income, highly transient population impaired our ability to recontact participants post-intervention. As such, we had a low response rate for the two- to three-year follow-up survey because we were unable to locate many women, thus our results may be biased. Nonetheless, our results suggest that participation in WIN Network may have lasting impacts for participants as they reported that WIN Network assisted them with numerous health behaviors, coping and support assistance, contraception, and pregnancy spacing. Similar to the control population, many of the participants in WIN Network were not married. It is unknown what role, if any, other support partners played in supporting the women during pregnancy as this data is not available in vital statistics records; however, it could be examined in future studies. Finally, we utilized a convenience sample for our comparison group, thus there may be inherent bias related to participation in WIN Network that we are unable to control for. The effect of participation bias is difficult to predict, but women who are more likely to participate in a program like this may have been more likely to use or seek out services or support systems which could have biased results away from the null. However, we conducted a sensitivity analysis in an effort to control for potential baseline differences in mental illness or drug or alcohol abuse. Although women would not have been eligible to participate in WIN Network if they self-reported drug or alcohol abuse or serious mental illness, prior work suggests that pregnant women do not necessarily self-report or seek treatment for these problems [30–32]. Thus, it is possible that some women in WIN Network had serious mental illness and/or abused drugs or alcohol. Nonetheless, we still found a significant decrease in NICU admissions. These associations will need to be validated in a future study.

In conclusion, our study suggests that use of CHWs may prevent a significant proportion of NICU admissions and improve the health of minority women and their children. The target population of low-income, Black women in Detroit experience a disproportionate burden of poverty, stressors, diseases, health inequities, social isolation, and limited access to resources; all of which contribute to disproportionately high rates of preterm and low-birth weight deliveries. Building upon existing relationships and trust between CHWs with healthcare and social service organizations and the community, WIN Network links women between disconnected clinical and social services to address social determinants. Previous studies suggest that social support is an important factor influencing pregnancy outcomes and maternal health and it may be one of the mechanisms by which CHWs have a positive impact, but this requires further study [33, 34]. While the CHWs cannot literally change an individual’s circumstances, this analysis demonstrates the effectiveness of CHWs in transforming the way women view the opportunities to thrive within these conditions. Interventions that incorporate CHWs in prenatal care programs must target the multifactorial nature of adverse pregnancy outcomes and improve conditions contributing to women’s health in order to ultimately decrease the racial disparities in pregnancy outcomes.

## Supporting information

S1 Table Association between WIN Network participation and infant health outcomes after excluding controls with late or no prenatal care and controls with missing information about prenatal care and/or month prenatal care started. (DOCX)
