# Supplementary material for: Community health workers as change agents in improving equity in birth outcomes in Detroit
Source: PLoS One. 2023 Feb 14;18(2):e0281450. doi: 10.1371/journal.pone.0281450 (PMC9928129; doi:10.1371/journal.pone.0281450)
Supplement: S1 Table — (DOCX) [file pone.0281450.s001.docx]

**S1 Table. Association between WIN Network participation and infant health outcomes after excluding controls with late or no prenatal care and controls with missing information about prenatal care and/or month prenatal care started.**

|  | WIN Network  Participants  (N= 254) | Controls (N= 9682) |  | |  | |  | |
| --- | --- | --- | --- | --- | --- | --- | --- | --- |
|  |  |  | p-value | OR (95% CI) | p-value | OR (95% CI) | p-value | OR (95% CI) |
| Low birth weight |  |  |  |  |  |  |  |  |
| Yes | 24 (9.5%) | 1057 (10.9%) | 0.458 | 0.85 (0.56, 1.30) | 0.410 | 0.84 (0.55, 1.28) | 0.511 | 0.86 (0.56, 1.34) |
| No | 230 (90.6%) | 8623 (89.1%) |  |  |  |  |  |  |
| Unknown | 0 (0%) | 2 (0%) |  |  |  |  |  |  |
| Preterm |  |  |  |  |  |  |  |  |
| Yes | 34 (13.4%) | 1514 (15.6%) | 0.328 | 0.83 (0.58, 1.20) | 0.311 | 0.83 (0.57, 1.19) | 0.430 | 0.86 (0.59, 1.25) |
| No | 220 (86.6%) | 8165 (84.3%) |  |  |  |  |  |  |
| Unknown | 0 (0%) | 3 (0%) |  |  |  |  |  |  |
| NICU |  |  |  |  |  |  |  |  |
| Yes | 15 (5.9%) | 951 (9.8%) | 0.033 | 0.57 (0.33, 0.96) | 0.033 | 0.57 (0.33, 0.96) | 0.031 | 0.55 (0.32, 0.95) |
| No | 239 (94.1%) | 8564 (88.5%) |  |  |  |  |  |  |
| Unknown | 0 (0%) | 167 (1.7%) |  |  |  |  |  |  |
| Infant mortality |  |  |  |  |  |  |  |  |
| Yes | 1 (0.4%) | 126 (1.3%) | 0.231 | 0.30 (0.04, 2.15) | 0.224 | 0.29 (0.04, 2.11) | 0.295 | 0.35 (0.05, 2.51) |
| No | 253 (99.6%) | 9556 (98.7%) |  |  |  |  |  |  |
|  | Mean ± SD | Mean ± SD | p-value | Mean difference  (95% CI) | p-value | Adjusted mean  difference (95% CI) | p-value | Adjusted mean  difference (95% CI) |
| Birth weight (grams) | 3145.3 ± 560.9 | 3113.8 ± 605.8 | 0.412 | 31.5 (-43.8, 106.9) | 0.317 | 38.4 (-36.9, 113.7) | 0.348 | 36.7 (-39.9, 113.3) |
| Gestational age (weeks) | 38.7 ± 2.8 | 38.4 ± 3.1 | 0.108 | 0.31 (-0.07, 0.69) | 0.115 | 0.31 (-0.07, 0.68) | 0.201 | 0.25 (-0.14, 0.64) |

OR, odds ratio; SD, standard deviation; WIN, Women-Inspired Neighborhood
